# miR-34a Regulates the Activity of HIF-1a and P53 Signaling Pathways by Promoting GLUT1 in Genetically Improved Farmed Tilapia (GIFT, *Oreochromis niloticus*) Under Hypoxia Stress

**DOI:** 10.3389/fphys.2020.00670

**Published:** 2020-06-16

**Authors:** Jun Qiang, Xiao-Wen Zhu, Jie He, Yi-Fan Tao, Jin-Wen Bao, Jun-Hao Zhu, Pao Xu

**Affiliations:** ^1^Fisheries College of Guangdong Ocean University, Zhanjiang, China; ^2^Key Laboratory of Freshwater Fisheries and Germplasm Resources Utilization, Ministry of Agriculture, Freshwater Fisheries Research Center, Chinese Academy of Fishery Sciences, Wuxi, China

**Keywords:** hypoxia stress, genetically improved farmed tilapia, miR-34a, GLUT1, apoptosis

## Abstract

In fish under hypoxia stress, homeostasis can become imbalanced, leading to tissue and organ damage and decreased survival. Therefore, it is useful to explore the molecular and physiological regulation mechanisms that function in fish under hypoxia stress. The microRNA miR-34a is involved in fat and glycogen metabolism, and in apoptosis. In this study, we first verified that *GLUT1*, the gene encoding glucose transporter 1, is a potential target gene of miR-34a in genetically improved farmed tilapia (GIFT, *Oreochromis niloticus*) by dual luciferase reporter assays. Then, we clarified the regulatory relationship between miR-34a and *GLUT1* by qRT-PCR analyses. We analyzed the regulatory effects of knockdown or promotion of *GLUT1* expression *in vitro* and *in vivo* in GIFT under hypoxia stress. The results confirm that *GLUT1* is a target gene of miR-34a in GIFT. Down-regulation of miR-34a significantly promoted *GLUT1* expression. Knockdown of *GLUT1* reduced the glycogen content in GIFT liver cells, inhibited *HIF-1a* gene expression, up-regulated the expression of genes involved in P53 signaling pathways (*P53* and *CASPASE-3* genes), and accelerated hepatocyte apoptosis under hypoxia stress. Compared with the control group, the group injected in the tail vein with miR-34a antagomir showed up-regulated expression of *GLUT1* in the liver, increased liver glycogen content at 96 h of hypoxia stress, down-regulated expression of *P53* and *CASPASE-3*, and decreased serum aspartate aminotransferase and alanine aminotransferase enzyme activities. Our results provide information about the molecular regulation mechanism of miRNAs and their target genes in fish during the response to hypoxia stress.

## Introduction

Oxygen is a key factor for animals at the cellular level. Without oxygen, oxidative metabolism, especially oxidative phosphorylation in the mitochondria, cannot occur (Trayhurn and Alomar, 2015). Exposure to hypoxia results in a series of complex physiological changes as the animal adapts to such conditions. The biological processes related to hypoxia are mainly mediated by the expression of genes in the hypoxia-inducing factor (HIF) signal pathway (Zhu et al., 2013), such as those encoding prolyl 4-hydroxylase (PHD), glucose transporters (GLUTs), Bcl2/adenocarcinoma E1B19KD interacting protein 3 (BNIP3), and the tumor suppressor protein P53 (P53). HIF is the main regulator of oxygen homeostasis. Under normoxic conditions, HIF is rapidly degraded through the VHL-dependent ubiquitin proteasome pathway, but HIF protein accumulates under hypoxic conditions (Ivan et al., 2001). Up-regulation of HIF can activate expression of its downstream target gene, *GLUT* (Shen et al., 2012). Yang et al. (2017) reported that under hypoxia stress, when there is insufficient oxygen to maintain normal physiological or behavioral functions, a series of physiological regulation activities are initiated in fish as they adapt to the conditions (Semenza, 2003; Ton et al., 2003). These changes lead to an increase in the number of GLUTs on the cell membrane. In Atlantic cod (*Gadus morhua*) exposed to hypoxia stress, *GLUT1* expression in gills was up-regulated, but both *GLUT1* and *GLUT3* were significantly down-regulated in the spleen (Hall et al., 2005). In cobia (*Rachycentron canadum*), the abundance of *GLUT1*, *GLUT3*, *GLUT5*, and *GLUT9* in the liver was higher in fish under hypoxia stress than in those in the control group (Wang et al., 2019). Zhang et al. (2003) detected up-regulated *GLUT* expression in the kidney and gill tissues of grass carp (*Ctenopharyngodon idellus*) under hypoxia stress. Zhao et al. (2020) detected up-regulated *GLUT1* mRNA expression in the muscle of largemouth bass (*micropterus salmoides*) under hypoxia stress. These findings indicate that the metabolic transition from aerobic oxidation to the glycolytic pathway is an effective strategy to increase glucose uptake to promote adaptation to hypoxic conditions (Ton et al., 2003).

MicroRNAs are a class of endogenous non-coding single-stranded small-molecule RNAs. They are ideal regulators of the oxygen load response because they can quickly and reversibly modify gene expression (Kulshreshtha et al., 2007; Pocock, 2011). In a previous study, many miRNA–mRNA interaction pairs were found in hypoxia-affected blunt snout bream (*Megalobrama amblycephala*), in which miR-210, miR-21, miR-17, and miR-29 were differentially expressed under hypoxia stress (Sun et al., 2017). Other studies showed that these miRNAs may regulate the expression of genes involved in the P53 signaling pathway (*P53* and *CASPASE-3*), thereby participating in the regulation of mitochondrial function, apoptosis, cell cycle arrest, and cell survival (Koumenis et al., 2001; Sun et al., 2016; Opdenbosch and Lamkanfi, 2019). In largemouth bass under hypoxia stress, miR-124 was shown to regulate lactate transportation in the muscle by targeting monocarboxylate transporter 1 (Zhao et al., 2020). miRNA transcriptomic analyses revealed that miR-34a in genetically improved farmed tilapia (GIFT) may be involved in the apoptosis pathway or in the regulation of glucose and lipid metabolism (Tao et al., 2017). In mammals, miR-34a is involved in a variety of pathophysiological processes, such as fatty acid oxidation and synthesis, fat and cholesterol metabolism, and apoptosis. miR-34a may be related to the occurrence and development of non-alcoholic fatty liver disease (NAFLD) (Yamakuchi et al., 2008). Studies have shown that miR-34a can regulate the expression of its target gene encoding silent information regulator 1, increase peripheral insulin resistance, regulate liver lipid metabolism, improve oxidative stress and mitochondrial dysfunction, and reduce inflammation, thus playing an important role in decreasing the symptoms of NAFLD (Nassir and Ibdah, 2016). During the proliferation of rat pulmonary artery smooth muscle cells (PASMC) induced by hypoxia, miR-34a was found to up-regulate the gene encoding NOTCH1, thereby participating in the control of PASMC proliferation (Ma et al., 2019). In blunt snout bream, miR-34a was shown to effectively up-regulate the gene encoding SIRTUIN 1 in the liver, thereby regulating the intake and metabolism of dietary fats and sugars (Miao et al., 2017).

Following on from studies on miR-34a in mammals, increasing attention has been paid to the role of miR-34a in fish. miR-34a is highly conserved among different species and may perform the same or similar functions in different vertebrates. Tilapia is one of China’s major freshwater farmed fish, and is the main aquacultured fish worldwide. High temperatures in summer, overfeeding, and nutritional imbalances can cause the water quality to deteriorate, and the dissolved oxygen (DO) content in pond water to drop sharply (Oliva-Teles, 2012; Qiang et al., 2019). Therefore, farmed tilapia are often subjected to hypoxia stress (Yuan et al., 2019). Previously, we identified that the tilapia *GLUT1* gene may be a potential target of miR-34a (score 149; free energy −24.48 Kcal/mol) (Tao et al., 2017). Therefore, the main aims of this study were to: (1) verify that *GLUT1* is a potential target gene of miR-34a; (2) analyze the effect of *GLUT1-*knockdown on the HIF-1a and p53 signaling pathway in GIFT liver cells; and (3) investigate how inhibition of miR-34a expression affects the response of GIFT to hypoxia stress. Studying the regulatory mechanism of miRNA and its target gene *GLUT1* in fish under hypoxia stress will be helpful for understanding physiological adaptation to hypoxic conditions.

## Materials and Methods

### Analysis of Binding Site and Regulatory Relationship for miRNA-Target Gene

The full-length 3′-untranslated (UTR) sequence of GIFT *GLUT1* (NM_001279727) was synthesized and inserted downstream of the luciferase gene in the pGL3-control vector. A mutation vector was constructed to mutate the target gene sequence CACTGCC into GTACAAT (mut). Cells (293T) were cultured at 37°C under 5% CO_2_. The 293T cells were seeded at a concentration of 1.5 × 10^4^/well into a 96-well plate with a volume of 100 μl per well and cultured at 37°C for 24 h. According to the Lipofectamine 2000 transfection procedure, the wild-type and mutant reporter plasmids were co-transfected with mimics (endogenous mature miRNA functional activity enhancer) and a mimic negative control (NC). Renilla luciferase was used to standardize the activity of each transfection well. At 36 h after transfection, cells were washed with pre-chilled PBS and collected by centrifugation at 800 × *g* for 5 min at 4°C. A liquid scintillation counter (LSC-7400, Hitachi, Tokyo, Japan) was used to detect luciferase activity.

Healthy GIFT (12.6 g ± 0.5) were selected, with 20 fish in each treatment group and three groups per treatment. The synthesized miR-34a antagomir and NC fragments (RiboBio, Guangzhou, China) were dissolved in PBS (5 nmol miRNA antagomir or NC fragment in 50 mL PBS) and were each injected into the tail of GIFT at a dose of 50 mg/kg body weight (25 μL reagent per fish). In the negative and blank control groups, a negative control (NC, 5′-UUUGUACUACACAAAAGUACUG-3′: no homology with the tilapia genome) and a PBS blank control were injected into tail vein at the same dose (Qiang et al., 2017a). Livers were collected from three GIFT per group at 0, 6, 12, 24, and 48 h after treatment. The GIFT were maintained in well-aerated water and treated with 200 mg/L tricaine methanesulfonate (Sigma, St Louis, MO, United States) for rapid deep anesthesia. After rapid freezing in liquid nitrogen, tissues were stored at −80°C until use.

### *GLUT1*-Knockdown in GIFT Liver Cells

The construction of the GIFT *GLUT1* RNA interference (RNAi) expression vector comprised the following steps: first, based on the tilapia *GLUT1* gene sequence (NM_001279727) in NCBI, four RNAi sequences were designed using BLOCK-iT^TM^ RNAi Designer. The sequences were confirmed to have no homology with other coding sequences in tilapia by BLAST analysis. After verification, an siRNA sequence (CCTCAGAAGATTATTGAGAACTTCA) with the best inhibition efficiency was selected. The oligonucleotide strand was annealed to form a double strand, ligated to a psiRNA vector digested with *Bbs*I, transformed into *Escherichia coli* GT116, and stored. The interference plasmid was named psiRNA-GLUT1, the NC interference plasmid was named psiRNA-Scramble, and the control plasmid was named psiRNA (-). Before transfection, hepatocytes were seeded in 12-well plates and divided into an experimental group, a NC group, and a control group, with 12 replicates per experimental group. When the cell concentration reached 80%, lipofectamine 2000 was used to transfect the RNAi expression vector into hepatocytes. The transfected hepatocytes were kept in an incubator (Hangzhou, China) for 24 h under hypoxic conditions (93% N_2_, 2% O_2_, 5% CO_2_). Six experimental wells were selected for each experimental group. The cells were lysed for 30 min and centrifuged at 4°C for 20 min, and then the supernatant was taken for gene expression analysis. The cells (1 × 10^6^ cells) in the other six duplicate wells of each experimental group were collected by centrifugation (3000 r/min, 5 min), mixed with 70% ethanol, and kept in an ice bath overnight. After passing through a 40 μm cell mesh sieve, the cells were incubated with 50 μg/mL propidium iodide, 100 μg/mL RNase, and 0.1% Triton X-100 at room temperature for 30 min in the dark, and then detected and analyzed with the Incyte module of an FACSCalibur flow cytometer (BD Biosciences, San Jose, CA, United States) (detection, 488 nm; emission 630 nm).

### Inhibition of miR-34a Expression in GIFT by Injection of miRNA Antagomir

A total of 360 GIFT juveniles (9.6 g ± 0.5) were randomly placed into nine 600-L tanks, each of which had 40 fish. Each treatment had three replicates. The synthesized miR-34a antagomir and NC fragments were each injected into the tail of GIFT at a dose of 50 mg/kg body weight (20 μL reagent per fish) (Yuan et al., 2019). At 12 h after injection, GIFT were placed into a hypoxic environment (DO of 1.1 ± 0.04 mg/L). Real−time readings with a DO meter (LDO101 probe, range 0.1–20.0 mg/L, Hach, Loveland, CO, United States) were used monitor the DO content in water, and the DO content was adjusted by altering the nitrogen and air charge. At the beginning of the experiment, the DO level in the experimental tanks was rapidly reduced over 1 h by pumping nitrogen (100 m^3^/h) into water from a nitrogen gas cylinder (Wuxi Guangming Special Gas Co., Ltd., Wuxi, China). Four fish were randomly selected from each tank at 0, 12, 24, 48, and 96 h after injection. After rapid deep anesthesia, blood was drawn from the tail vein using a 1.5-ml syringe. Blood samples were kept at 4°C for 2 h, then centrifuged at 3000 × *g* at 4°C for 10 min. The serum was collected and placed stored at −80°C until analysis of serum alanine aminotransferase (ALT) and aspartate aminotransferase (AST) activities. At the same time, liver tissues were sampled, rapidly frozen in liquid nitrogen, and then stored at −80°C until analyses of the miR-34a regulatory response mechanism.

### Real-Time Quantitative PCR for miRNA and mRNA

Total RNA was extracted from the samples according to the instructions of the Trizol reagent kit. Total RNA purity and the concentration of RNA were determined using Agilent 2100 (Agilent Technologies, CA, United States) and NanoDrop 8000 (NanoDrop, United States) instruments (RNA integrity number was >7.0; 28S/18S ≥ 1.5). Reverse transcription of miRNA cDNA was performed using the Mir-X^TM^ miRNA First-Strand Synthesis Kit (TaKaRa, Dalian, China). The miRNA primers were synthesized by GENEWIZ Inc. (Suzhou, China) (Table 1). For qRT-PCR amplification of miRNAs, the reaction mixture contained 10 μL 2 × SYBR^®^ Premix Ex Taq^TM^ II (TaKaRa), 0.4 μL 50 × ROX reference dye, 0.4 μL each of upstream and downstream primers (10 μmol/L), 2 μL miRNA-cDNA template, and ddH_2_O to complete the volume to 20 μL. We used U6 as a reference gene for miRNA quantification. Three replicates were analyzed for each sample, and non-templated cDNA was used as the negative control. The thermal cycling conditions were as follows: 95°C for 30 s, 95°C for 5 s, and 60°C for 31 s (40 cycles). The dissolution profile of the amplified product was analyzed at the end of each PCR cycle. After amplification, the temperature was raised from 60°C, and the specificity of the amplification product was verified from the dissolution curve.

**TABLE 1:** Sequences of primers used for qRT-PCR.

Name	Primer sequence (5′-3′)	Registration numbers
GLUT1	F: 5′- TAACCGCTTGGGAAGGAGGA-3′ R: 3′- CCAACCACAAAACGGCCAAT-5′	FJ914657.1
HIF-1a	F: 5′- GCACAGTTTGACTTGACTGGAC-3′ R: 5′- TTCTTGGAGCCTGTTCTGTGG-3′	KY415998.1
P53	F: 5′-TTTTCTCCTCCCTGTTCGTGG-3′ R: 5′- CGGGAACCTCATGCTTCACT-3′	GU594898.1
CASPASE-3	F: 5′- GAAACGAACAGCAGCAGACC-3′ R: 5′- CGAGTGCTCATCCCTGTTGT-3′	GQ421464.1
18S rRNA	F: 5′-GGCCGTTCTTAGTTGGTGGA-3′ R: 5′-TTGCTCAATCTCGTGTGGCT-3′	U67340.1
miR-34a	TGGCAGTGTCTTAGCTGGTTGT	

The RT reaction and qRT-PCRs of the mRNAs were conducted using PrimeScript^TM^ RT Master Mix and SYBR^®^ Premix Ex Taq kits (TaKaRa), as described previously (Qiang et al., 2017a). 18S rRNA was used as the reference gene. The mRNA and 18S rRNA primers were synthesized by Shanghai GeneCore BioTechnologies Co., Ltd. (Shanghai, China). The levels of miRNA and mRNA were calculated using the 2^–ΔΔCT^ method, and analyzed with Relative Quantification Manager software included with the 7900HT Fast Real-Time PCR System (Applied Biosystems, Foster City, CA, United States).

### Analysis of Serum Biochemical Indexes and Enzyme Activities

The collected hepatocytes were mixed with PBS, ultrasonically disrupted, and then the glycogen content was determined as described by Frutos et al. (1990). The assay kit was provided by the Nanjing Jiancheng Biotechnology Research Institute (Nanjing, China). The serum ALT and AST activities and hepatic glycogen content were determined by enzyme-linked immunosorbent assay (ELISA) using test kits purchased from Lexington Biosciences (Shanghai, China).

### Statistical Analyses

The results are expressed as mean ± standard deviation (mean ± SD). Experimental data were subjected to analysis of variance using SPSS 21.0 statistical software (SPSS Inc., Chicago, IL, United States). First, the data were tested for a normal distribution and homogeneity of variance. Independent samples *t*-test was used to compare values among different treatment times within the same experimental group; Duncan’s multiple comparison was used to compare different treatment groups at the same time. Differences were considered significant at *P* < 0.05.

## Results

### Identification of Binding Site and Regulation Relationship Between miR-34a and Its Potential Target Genes

**FIGURE 1:**
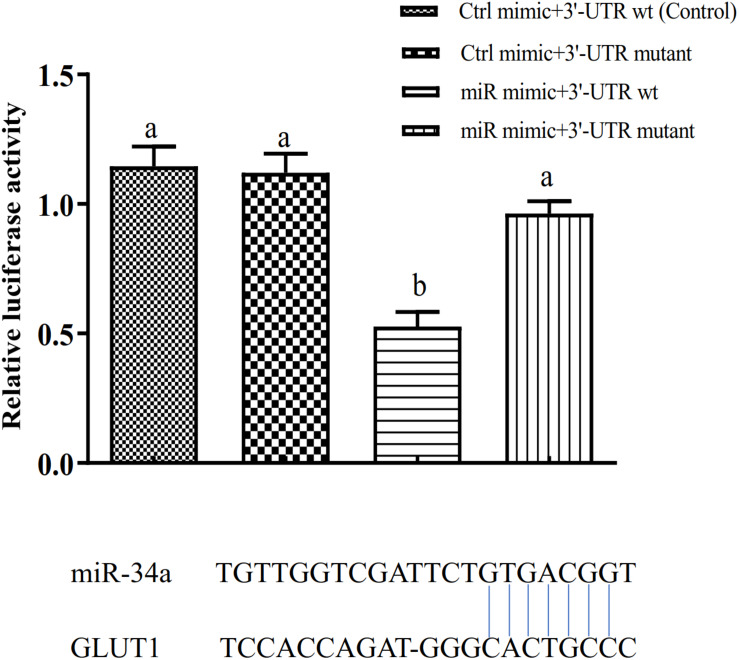
Verification of binding site of miRNA to potential target gene using dual luciferase reporter system (*n* = 8). HEK-293T cells in 96-well plates were co-transformed with the construct pGL-GLUT1 3′ UTR (WT) or pGL-GLUT1 (Mut) and miRNA mimic or miRNA negative control using Lipofectamine 2000 transfection reagent. Different lowercase letters indicate significant differences among experimental groups (Duncan’s multiple comparison; *P* < 0.05).

**FIGURE 2:**
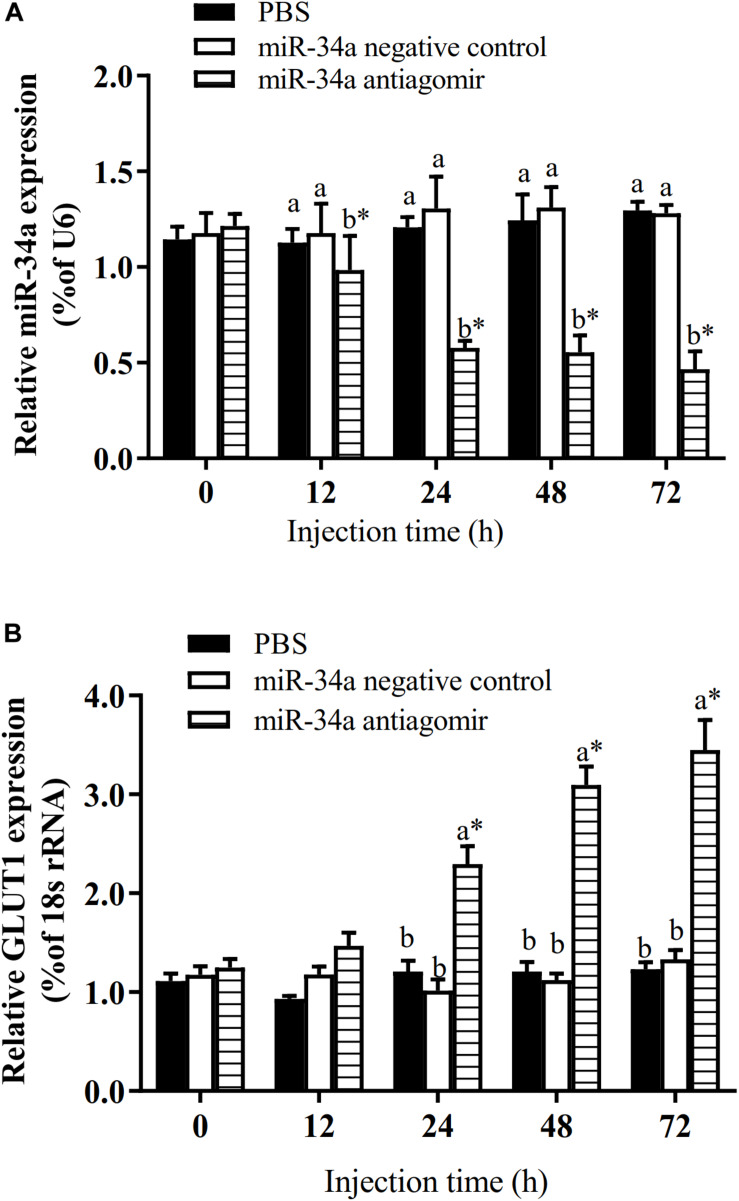
Analysis of regulatory relationships between miR-34a **(A)** and *GLUT1* **(B)** *in vivo* (*n* = 9). Juvenile GIFT weighing about 12.6 ± 0.5 g were injected in tail vein with miR-34a negative control, or miR-34a antagomir (dose, 50 mg/kg body weight) or with PBS (control). *Indicates significant differences between values obtained before and after injection (paired-samples *t*-test; *P* < 0.05). Different lowercase letters show significant differences among different treatments at each sampling point (Duncan’s multiple range test; *P* < 0.05).

The target gene validation results showed that the miR-34a mimic significantly down-regulated the *GLUT1* wild-type reporter fluorescence, while the fluorescence in the treatment with the mutated predicted target site was not significantly different from that in the control (*P* < 0.05). This result indicated that miR-34a regulates the expression of its target genes through the site that was mutated in the mut vector (see Figure 1). A sequence in the *GLUT1* 3′-UTR region is completely complementary to positions 2–8 of the miR-34a 5′ end seed region. To further verify the targeted regulation effect of miR-34a on *GLUT1*, we injected miR-34a antagomir into the tail vein of GIFT. The results showed that as the expression level of miR-34a decreased, the level of *GLUT1* mRNA increased significantly (*P* < 0.05, Figure 2).

### Effect of *GLUT1* Knockdown on Expression of *HIF-1a*, *P53* and *CASPASE-3*, and Proportions of Apoptotic Hepatocytes in GIFT Under Hypoxia Stress

**FIGURE 3:**
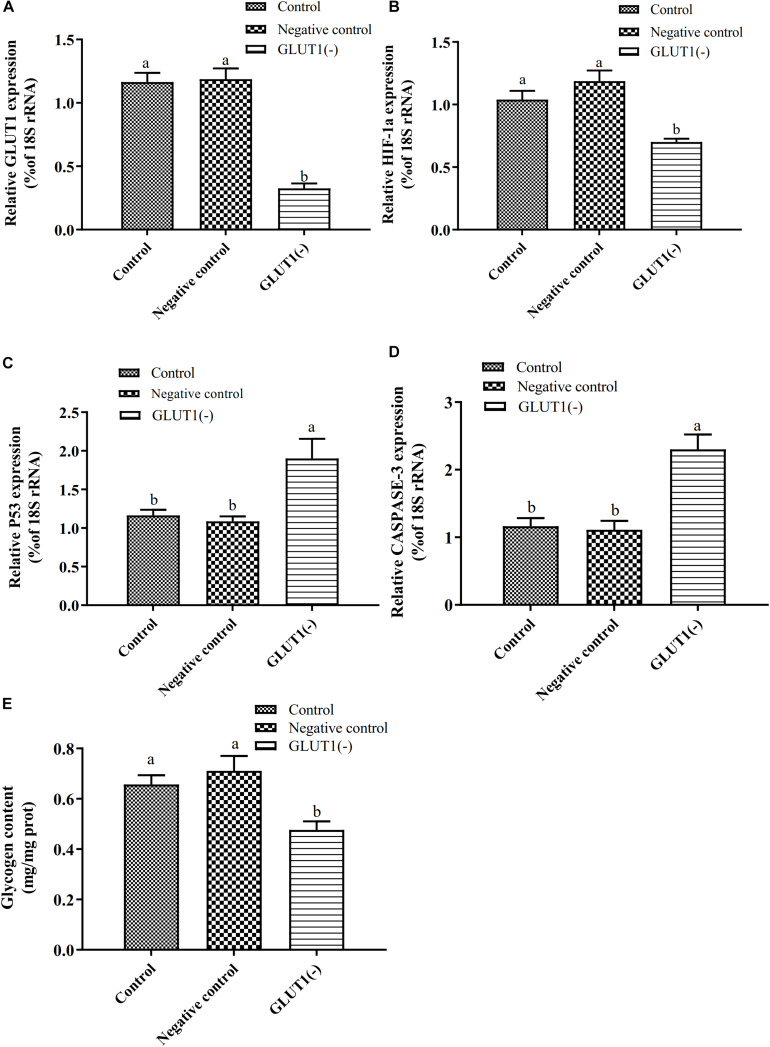
Effect of *GLUT1* knockdown on transcript levels of *GLUT1* **(A)**, *HIF-1a* **(B)**, *P53* **(C)**, and *CASPASE-3* **(D)**, and glycogen content **(E)** in GIFT hepatocytes under hypoxia stress (*n* = 6). Transcript levels in control, negative control, and GLUT1(-) groups are shown. Different lowercase letters indicate significant differences among experimental groups (Duncan’s multiple comparison; *P* < 0.05).

**FIGURE 4:**
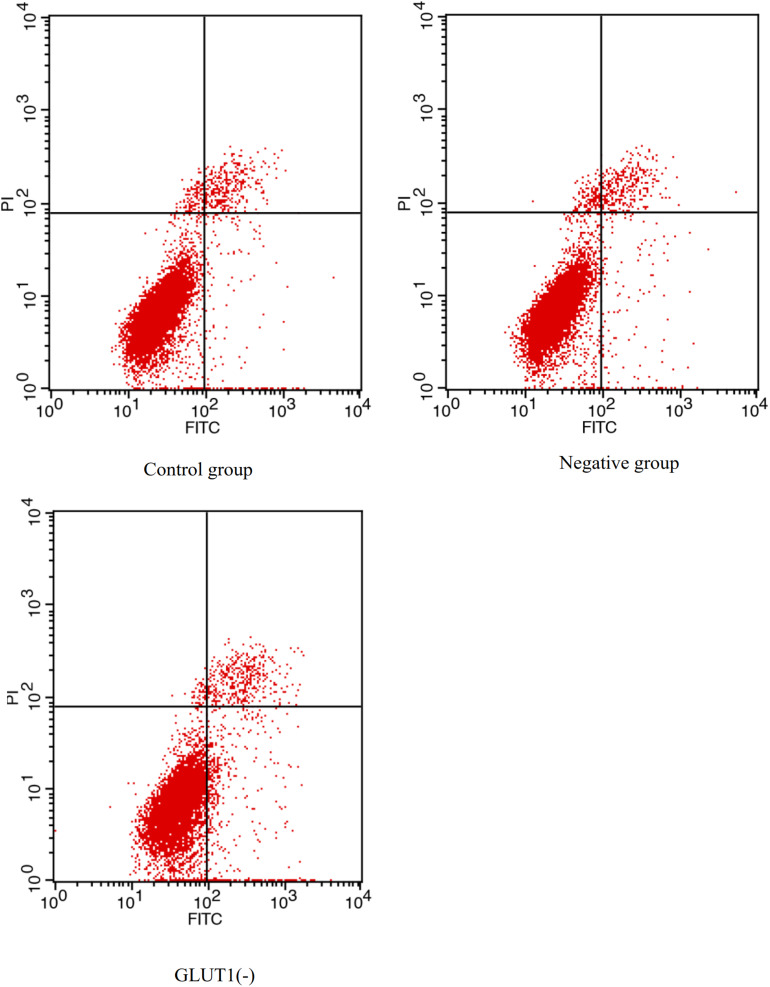
Effect of *GLUT1* knockdown on apoptosis in hepatocytes of GIFT under hypoxia stress (*n* = 6). Proportion of apoptotic cells in control, negative control, and GLUT1(-) groups are shown.

The *GLUT1* mRNA level was 65% lower (*P* < 0.05) in the *GLUT1*-knockdown group than in the control group and the *GLUT1*-NC transfection group. The *HIF-1a* mRNA level was significantly lower in the *GLUT1-*knockdown group than in the control group and the *GLUT1*-NC transfection group (Figure 3). However, the mRNA levels of the apoptotic pathway-related genes *P53* and *CASPASE-3* were significantly higher in the *GLUT1*-knockdown group than in the control group and the *GLUT1-*NC transfection group. The glycogen content in liver cells was also significantly lower in the *GLUT1-*knockdown group than in the control group and the *GLUT1*-NC transfection group. The flow cytometry analysis showed that, under hypoxia stress, the proportions of apoptotic hepatocytes were significantly lower in the control (6.61%) and *GLUT1*-NC transfected groups (6.48%) (Q4 quadrant) than in the *GLUT1-*knockdown group (10.96%) (Figure 4).

### Effect of Inhibition of miR-34a on Gene Expression, Glycogen Content, and Activities of AST and ALT in GIFT Under Hypoxia Stress

**FIGURE 5:**
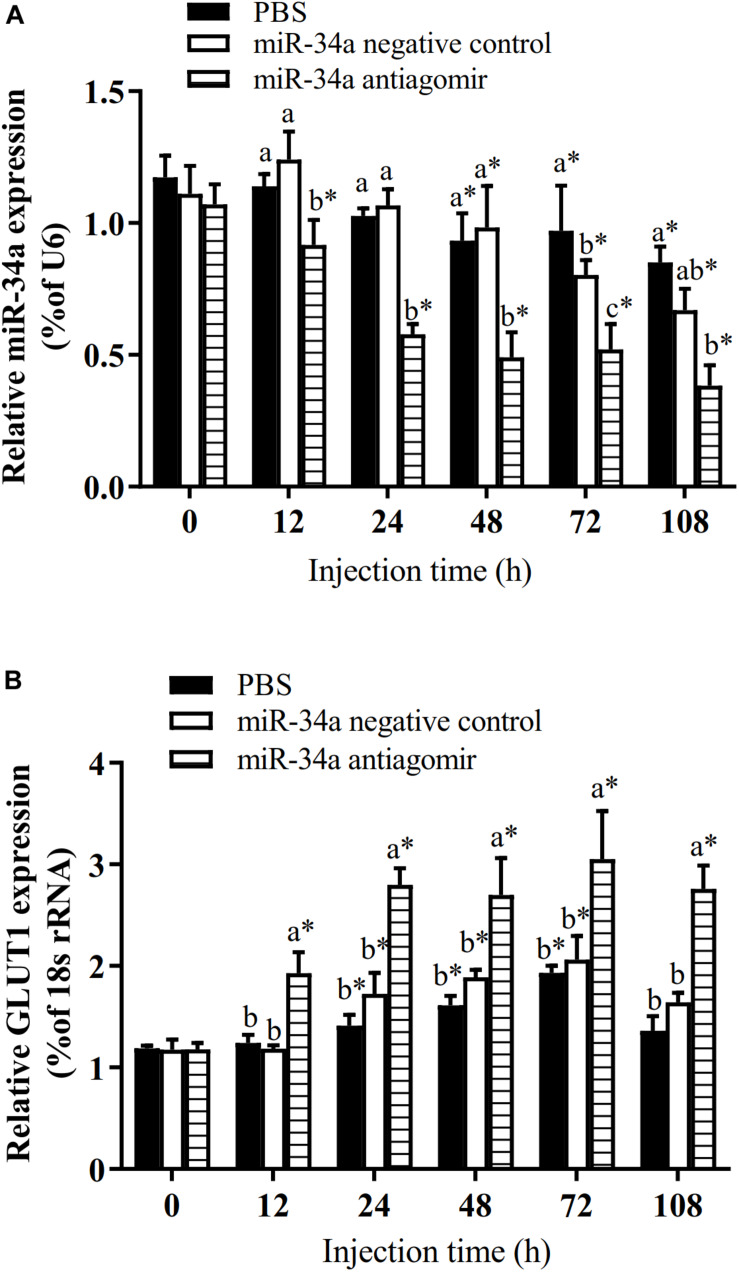
Expression levels of miR-34a **(A)** and *GLUT1* **(B)** in liver of GIFT with inhibited miR-34a expression (*n* = 12). Juveniles weighing about 9.6 ± 0.5 g were injected in tail vein with PBS, miR-34a negative control, or miR-34a antagomir (dose, 50 mg/kg body weight) and response was monitored for 108 h. *Indicates significant differences between values obtained before and after injection (paired-samples *t*-test; *P* < 0.05). Different lowercase letters indicate significant differences among different treatments at each sampling point (Duncan’s multiple comparison; *P* < 0.05).

To further analyze how miR-34a regulates target genes in the liver of GIFT during the response to hypoxia stress, we injected miR-34a antagomir into the tail vein. The level of *GLUT1* mRNA in liver tissues was significantly higher in the antagomir group than in the control group and NC group at 12, 24, 48, 72, and 108 h after injection; meanwhile, the expression level of miR-34a was significantly lower in the antagomir group than in the control and NC groups at each sampling time (Figure 5). At 12 h after miRNA antagomir injection, the GIFT were subjected to hypoxia conditions (DO of 1.1 mg/L). In the control group, the expression level of *GLUT1* in the liver increased first and then decreased during 96 h of hypoxia stress.

**FIGURE 6:**
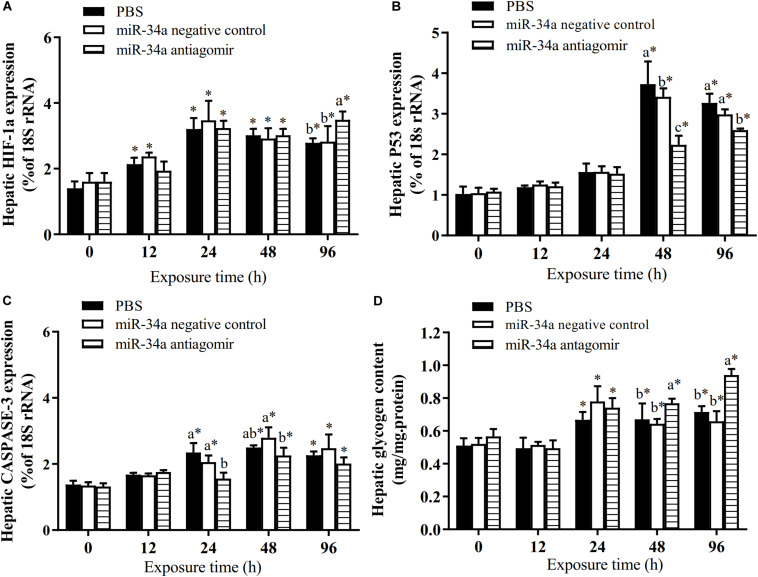
Transcript levels of genes encoding *HIF-1a* **(A)**, *P53* **(B)** and *CASPASE*-3 **(C)** in the liver and hepatic glycogen content **(D)** of GIFT with inhibited miR-34a expression (*n* = 12). At 12 h after miR-34a antagomir injection, juveniles were subjected to hypoxia stress for 96 h. GIFT injected with PBS served as control. *Indicates significant differences between values obtained before and after injection (paired-samples *t*-test; *P* < 0.05). Different lowercase letters indicate significant differences among different treatments at each sampling point (Duncan’s multiple comparison; *P* < 0.05).

**FIGURE 7:**
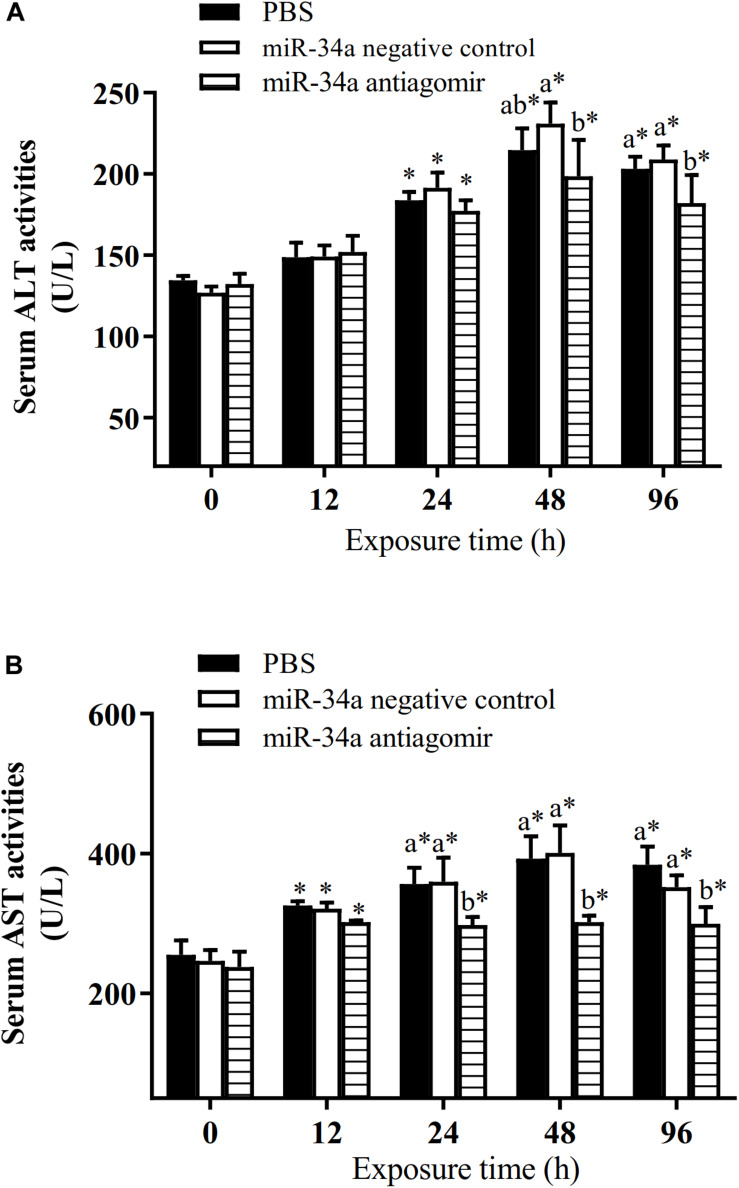
Activities of ALT **(A)** and AST **(B)** in serum of GIFT treated to inhibit miR-34a expression (*n* = 12). At 12 h after miR-34a antagomir injection, juveniles were subjected to hypoxia stress for 96 h. GIFT injected with PBS served as control. *Indicates significant differences between values obtained before and after injection (paired-samples *t*-test; *P* < 0.05). Different lowercase letters indicate significant differences among different treatments at each sampling point (Duncan’s multiple comparison; *P* < 0.05).

As shown in Figure 6, the mRNA levels of *P53* and *CASPASE-3* in the liver increased in the control group and the NC group under hypoxia stress. The liver *CASPASE-3* mRNA expression level was significantly lower in the antagomir group than in the control group and NC group at 24 h of hypoxia stress. The *P53* mRNA level was significantly lower in the antagomir group than in the control and NC groups at 48 and 96 h of hypoxia stress. In addition, in all groups, the mRNA levels of *caspase-3* and *p53* were significantly higher at 48 and 96 h of hypoxia stress than before the stress treatment. Similarly, the levels of *HIF-1a* mRNA in each treatment group were significantly higher at 24, 48, and 96 h of hypoxia stress than before the stress treatment. At 96 h of hypoxia stress, the *HIF-1a* mRNA levels were significantly higher in the antagomir group than in the control and NC groups. In all groups, the hepatic glycogen content decreased slightly at 12 h of hypoxia stress, and was significantly higher than pre-stress levels at 24, 48, and 96 h of hypoxia stress. The hepatic glycogen content was significantly higher in the antagomir group than in the control group and NC group at 48 and 96 h of hypoxia stress. In all groups, the serum ALT and AST activities tended to increase under hypoxia stress, and were significantly higher than pre-stress levels at 24, 48 and 96 h (Figure 7). After 24 h of hypoxia stress, the activity of AST was significantly lower in the antagomir group than in the control group and NC group. Also, the serum ALT activities were significantly lower in the antagomir group than in the control group and the NC group at 48 and 96 h of hypoxia stress.

## Discussion

### Inhibition of miR-34a Expression Promotes *GLUT1* Expression in GIFT

First, we confirmed that miR-34a can negatively regulate the expression of *GLUT1*. Recent studies have shown that inhibition of miR-34a expression can reduce cell death and fibrosis after acute myocardial infarction, and promote the recovery of myocardial function (Boon et al., 2013; Yang et al., 2015). Overexpression of miR-34a can promote apoptosis of the myocardial cell line H9C2 under hypoxia and reoxygenation or in a high blood glucose state (Zhao et al., 2013). In addition, inhibition of miR-34a expression in primary cultured neonatal rat cardiomyocytes can reduce cardiomyocyte apoptosis and improve cardiomyocyte viability under hypoxia–reoxygenation conditions, suggesting that miR-34a plays an important regulatory role in alleviating hypoxia stress and improving adaptation to such conditions (Fan et al., 2017). In our previous study, a cluster analysis revealed that the potential target genes of miR-34a in GIFT are concentrated in lipid metabolism and apoptosis signaling pathways (Tao et al., 2017). Therefore, in this study, we focused on how miR-34a regulates *GLUT1* in GIFT exposed to hypoxia stress.

### Knockdown of *GLUT1* Increases Apoptosis of Hepatocytes

To provide energy for cell metabolism, glucose needs to be transported from the outside to the inside of the cell via a glucose transporter on the cell membrane. GLUT1 is widely distributed in mammalian tissues and is a non-insulin-dependent glucose transporter (Szablewski, 2017). The expression of GLUT1 in cells and its redistribution within cells affect cell growth and apoptosis, and it is involved in the development of tissues and organs and tumor formation (Medina and Owen, 2002; Oh et al., 2017). *GLUT1* expression can be up-regulated by HIF-1a-mediated hypoxia response elements and promote the basic sugar supply to tissue cells (Yang et al., 2017). In hypoxia-sensitive cells, up-regulation of *GLUT* expression facilitates glucose transport (Semenza, 2003). In this study, the expression of HIF-1a in liver cells was inhibited in the *GLUT1*-knockdown group under hypoxia stress, resulting in decreased transfer of extracellular glucose into cells. Activation of P53 and CASPASE-3 can induce cell necrosis, apoptosis, or autophagy, all of which are involved in cell death. In the *GLUT1*-knockdown group, the expression of *P53* and *CASPASE-3* genes in GIFT liver cells was significantly up-regulated under hypoxia stress. Down-regulation of *GLUT1* may have led to an insufficient glucose supply and increased apoptosis in GIFT liver cells.

### Regulation Mechanisms of GIFT Injected With miRNA Antagomir During Hypoxia Stress

We found that hypoxia stress significantly activated *HIF-1a* mRNA expression in the liver tissues of GIFT. The HIF signaling pathway is a hot topic in research on the response of fish to hypoxia stress. Other studies have reported that Chinese sucker (*Myxocyprinus asiaticus*) (Chen et al., 2012), Indian catfish (*Clarias batrachus*) (Mohindra et al., 2013), and mudskipper (*Boleophthalmus pectinirostris*) (Ren et al., 2018) exposed to acute hypoxia show increased mRNA levels of HIF-related genes. However, in our study, the *HIF-1a* mRNA level in the liver was significantly higher in the antagomir group than in the control group at 96 h of hypoxia stress. Up-regulated *HIF-1a* mRNA levels may have activated downstream regulatory pathways to strengthen the fish body to respond to hypoxia stress.

miR-34a negatively regulates the expression of *HIF-1a*; consequently, injection of the miR-34a antagomir into GIFT significantly increased the *GLUT1* mRNA levels and increased the glycogen content in the liver. This may have helped to enhance energy supply during hypoxia stress. Similar results have been reported for largemouth bass, mudskipper, and carp (*Cyprinus carpio*) (Lardon et al., 2013; Yang et al., 2017; Ren et al., 2018). Our results show that in GIFT exposed to an acute hypoxic environment, expression of *HIF-1a* and its downstream target gene *GLUT1* is activated, leading to increased glucose uptake to maintain energy requirements.

Hypoxia-induced P53 signaling pathways can help to eliminate stress cells as an adaptation to hypoxia (Sun et al., 2016). In our study, the mRNA level of *P53* in the liver of the control group increased and then decreased during the 96 h of hypoxia stress. Under hypoxia stress, HIF activates Bcl-2 family members to promote the expression of *P53*, which is involved in anaerobic metabolism (Shimizu et al., 1996). Several studies have shown that the expression of *P53* is significantly up-regulated during hypoxia stress in aquatic animals such as oriental river prawn (*Macrobrachium nipponense*) (Kulshreshtha et al., 2007) and whiteleg shrimp (*Litopenaeus vannamei*) (Felix-Portillo et al., 2016). Because P53 functions as general receptor for environmental stress, its overexpression may lead to increased oxidative stress in cells and increased apoptosis. A previous study showed that silencing of P53 in white shrimp leads to increased expression of cyclin-dependent kinase 2, which functions in cell cycle progression, and decreased expression of CASPASE-3 under 48 h of hypoxia stress, and these changes regulate liver cell apoptosis and the cell cycle (Nuñez-Hernandez et al., 2018). Therefore, in the later stage of hypoxia stress, the down-regulated expression of *P53* in the control group may represent a hypoxia protection mechanism in GIFT. We found that down-regulation of miR-34a in the antagomir group led to up-regulation of *GLUT1*, thereby enhancing the intracellular energy supply and alleviate liver stress.

Apoptosis is a process in which multicellular organisms regulate their own growth, development, and immune response. According to their role in the apoptotic signaling pathway, apoptosis-related CASPASEs can be further divided into initial CASPASEs (2, 8, 9, 10) and effect CASPASEs (3, 6, 7) (Opdenbosch and Lamkanfi, 2019). Apoptosis-related CASPASEs are widely expressed in various fish tissues, with relatively high expression levels in immune-related tissues (Laing et al., 2001). In our study, the *CASPASE-3* mRNA level in liver tissues significantly increased in GIFT under hypoxia stress, suggesting that the CASPASE signaling pathway may also play an important role in hypoxia adaptation in fish. Reis et al. (2007) detected low expression of *CASPASE-3* in healthy sea bass (*Dicentrarchus labrax*), but an increase in *CASPASE-3* expression after infection with a photobacterium. In large yellow croaker (*Pseudosciaena crocea*) challenged with poly I: C and bacteria, the expression of *CASPASE-3* in the kidney and spleen was found to significantly increase, which may have activated the intrinsic apoptosis pathway (Li et al., 2011). However, in the present study, up-regulation of *GLUT1* in the antagomir group slowed the increase in *CASPASE-3* expression, which may have alleviated apoptosis of GIFT liver cells. Previous studies have shown that the down-regulation of miR-532-5p in myocardium H9c2 cells and in myocardial infarction-affected rats under hypoxia stress can up-regulate the expression of its target gene encoding programmed cell death protein 4, and promote the expression of *CASPASE-3*, thereby increasing H9c2 apoptosis (Ma et al., 2018). In addition, inhibition of miR-9 can up-regulate the expression of the gene encoding Yes-associated protein 1, promote cell proliferation, and inhibit CASPASE-3/7 activity and apoptosis in hypoxic H9c2 cells (Zheng et al., 2019).

Serum biochemical indexes reflect changes in the body’s metabolism and the function of tissues and organs during environmental stress in animals. The DO levels significantly affect blood biochemical indexes in farmed fish (Qiang et al., 2017b). Under healthy conditions, ALT and AST are mainly found in the liver, and only small amounts are released into the blood. Previously, we found that the serum ALT and AST activities of GIFT significantly increase under hypoxia stress (Yuan et al., 2019). Similar results were found in this study. In GIFT, hypoxia may cause liver damage, increase the permeability of the liver cell membrane, induce the release of ALT and AST from liver cells, and increase their enzyme activity in serum. However, the up-regulation of *GLUT1* in the antagomir group may have helped to alleviate liver cell damage, which would explain the decrease in serum ALT and AST activities.

In summary, our results show that silencing of miR-34a in GIFT increases glucose transport and interferes with the hypoxia-induced apoptotic pathway (via down-regulation of *P53* and *CASPASE-3* mRNA expression levels) by targeting *GLUT1*. Ultimately, these changes alleviate liver damage and reduce serum ALT and AST activities. Therefore, adjustment of glucose transport by miRNA or genetic manipulation may contribute to relieving stress and/or treating fish under hypoxia stress. In this study, we explored the regulatory function of miR-34a and its target gene *GLUT1* only at the gene expression level. In further studies, we intend to analyze the regulatory pathways related to GLUT1 at the -omics and protein levels.

## Data Availability Statement

The raw data supporting the conclusions of this article will be made available by the authors, without undue reservation, to any qualified researcher.

## Ethics Statement

The study protocols and design were approved by the Ethics Committee at the Guangdong Ocean University (Zhanjiang, China). The GIFT were maintained in well-aerated water and treated with 200 mg/L tricaine methanesulfonate (Sigma, St. Louis, MO, United States) for rapid deep anesthesia. The samples were extracted based on the Guide for the Care and Use of Laboratory Animals in China.

## Author Contributions

X-WZ and PX conceived and designed the experiments. JQ isolated and cultured the tilapia liver cells. JQ and Y-FT verified the intracellular function of miRNA and analyzed the data. JH, Y-FT, and J-WB conducted the hypoxia stress experiments and collected the samples, extracted RNA, and conducted the qRT-PCR experiments. J-HZ conducted the biochemical analyses. JQ and X-WZ wrote the manuscript with contributions from all other authors. All authors read and approved the final version of the manuscript.

## Conflict of Interest

The authors declare that the research was conducted in the absence of any commercial or financial relationships that could be construed as a potential conflict of interest.
